# Floral Scent Mimicry and Vector-Pathogen Associations in a Pseudoflower-Inducing Plant Pathogen System

**DOI:** 10.1371/journal.pone.0165761

**Published:** 2016-11-16

**Authors:** Scott H. McArt, Timothy D. Miles, Cesar Rodriguez-Saona, Annemiek Schilder, Lynn S. Adler, Matthew J. Grieshop

**Affiliations:** 1 Department of Entomology, Cornell University, Ithaca, New York, 14853, United States of America; 2 School of Natural Sciences, California State University–Monterey Bay, Seaside, California, 93955, United States of America; 3 Department of Entomology, Philip E. Marucci Center, Rutgers University, Chatsworth, New Jersey, 08019, United States of America; 4 Department of Plant, Soil and Microbial Sciences, Michigan State University, East Lansing, Michigan, 48824, United States of America; 5 Department of Biology, University of Massachusetts, Amherst, Massachusetts, 01003, United States of America; 6 Department of Entomology, Michigan State University, East Lansing, Michigan, 48824, United States of America; Indian Institute of Science, INDIA

## Abstract

Several fungal plant pathogens induce ‘pseudoflowers’ on their hosts to facilitate insect-mediated transmission of gametes and spores. When spores must be transmitted to host flowers to complete the fungal life cycle, we predict that pseudoflowers should evolve traits that mimic flowers and attract the most effective vectors in the flower-visiting community. We quantified insect visitation to flowers, healthy leaves and leaves infected with *Monilinia vaccinii-corymbosi* (*Mvc*), the causative agent of mummy berry disease of blueberry. We developed a nested PCR assay for detecting *Mvc* spores on bees, flies and other potential insect vectors. We also collected volatiles from blueberry flowers, healthy leaves and leaves infected with *Mvc*, and experimentally manipulated specific pathogen-induced volatiles to assess attractiveness to potential vectors. Bees and flies accounted for the majority of contacts with flowers, leaves infected with *Mvc* and healthy leaves. Flowers were contacted most often, while there was no difference between bee or fly contacts with healthy and infected leaves. While bees contacted flowers more often than flies, flies contacted infected leaves more often than bees. Bees were more likely to have *Mvc* spores on their bodies than flies, suggesting that bees may be more effective vectors than flies for transmitting *Mvc* spores to flowers. Leaves infected with *Mvc* had volatile profiles distinct from healthy leaves but similar to flowers. Two volatiles produced by flowers and infected leaves, cinnamyl alcohol and cinnamic aldehyde, were attractive to bees, while no volatiles manipulated were attractive to flies or any other insects. These results suggest that *Mvc* infection of leaves induces mimicry of floral volatiles, and that transmission occurs primarily via bees, which had the highest likelihood of carrying *Mvc* spores and visited flowers most frequently.

## Introduction

Plant pathogens can induce changes in their hosts, influencing the frequency and nature of interactions with animal vectors [1]. Pathogen manipulation of host plant phenotypes is increasingly described in plant-herbivore-pathogen systems [2–6], and we are beginning to understand the prevalence of such manipulation in plant-pollinator-pathogen systems. For example, of 26 plant pathogenic species of fungi, bacteria and viruses known to be vectored by floral visitors, at least 10 manipulate their hosts to increase the likelihood of transmission [7, 8].

Pollinator-vectored plant pathogens can manipulate their hosts in two ways. First, they can induce changes in host floral traits to increase chances of transmission. For example, the pathogens *Microbotryum violaceum* and *Fusarium verticillioides* cause flowers from their host plants to bloom earlier and stay open longer than healthy plants, leading to increased visitation from both invertebrate and vertebrate vectors [9–11]. A more aggressive tactic used by some fungal plant pathogens involves the production of ‘pseudoflowers’ from host plant vegetative tissue. In such cases, plants become infected during vegetative growth and produce pathogen-induced flower-like structures in place of or during host flowering. Insects visit these structures and facilitate either sexual outcrossing of the fungus by visiting other pseudoflowers [12–18], or transmission of infective spores to flowers of the host plant [19–21].

When vectors facilitate sexual outcrossing of pseudoflower-inducing fungi, there is little reason to expect trait mimicry between pseudoflowers and host flowers. Instead, to avoid unnecessary loss of gametes to co-blooming host flowers, natural selection would be expected to favor pseudoflower phenotypes that are distinct from host flower phenotypes. The few examples we have to date support this hypothesis. For example, the pseudoflower structures induced by *Puccinia monoica*, *P*. *arrhenatheri* and *Uromyces pisi* ‘bloom’ at different times than flowers of their respective hosts, *Arabis drummondii*, *Berberis vulgaris* and *Euphorbia cyparissias* [12–14, 22]. Furthermore, pseudoflower scent is distinct from that of host flowers. Raguso and Roy (16) found that *P*. *monoica* pseudoflower fragrance consisted entirely of aromatic alcohols, aldehydes and esters, while host floral volatiles consisted of terpenoids and aliphatic green leaf volatiles (GLVs). Similarly, Naef et al. [14] found that only two minor headspace constituents, 6-methyl-5-heptane-2-one and 2-phenylethanol, were shared between *P*. *arrhenatheri* pseudoflowers and *Berberis vulgaris* host flowers; all other volatiles were distinct.

When vectors facilitate transmission of pseudoflower-inducing fungi to host flowers, pseudoflower traits would be expected to mimic host floral traits to encourage co-visitation to both pseudoflowers and host flowers. However, evidence of such trait mimicry has remained largely anecdotal [20]. Furthermore, for most pseudoflower-inducing fungi, the links between disease-induced traits, vector attraction and transmission are poorly understood. Such knowledge is important not only for understanding the evolutionary ecology and transmission biology of plant-pollinator-pathogen systems, but also for disease management. For example, vectored plant pathogens that manipulate host traits are responsible for some of the most economically devastating agricultural problems, such as bacterial wilt in cucurbits [23], citrus greening disease [4, 24] and mummy berry disease in blueberry [19, 25]. In order to make informed management decisions regarding disease control, an understanding of disease-induced traits, vectors and transmission is essential.

**Fig 1:**
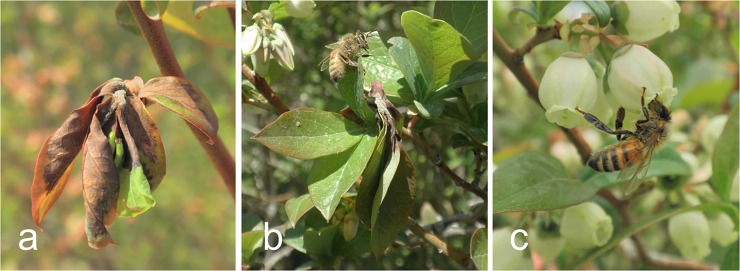
Blueberry leaves infected with *Monilinia vaccinii-corymbosi* (*Mvc*) exhibit fungal conidia in a sugary matrix on the surface of blighted tissue, called mummy berry shoot ‘strikes’ (A and B). Honey bee foraging on a blueberry flower (C).

Here, we use *Monilinia vaccinii-corymbosi* (*Mvc*), an Ascomycete fungal pathogen that causes mummy berry disease in blueberry (*Vaccinium corymbosum*), to understand how pseudoflower mimicry and vector-pathogen associations within the flower visiting community potentially shape disease transmission. Mummy berry disease has two distinct phases. Primary infection occurs when aerially dispersed ascospores produced in apothecia on overwintered fruit mummies infect newly expanding shoots and lead to blighting of vegetative tissue [Fig 1A, 19]. Fungal conidia are produced in a sugary matrix on the surface of blighted tissue (called ‘strikes’), which are visited by insects during the normal flowering period for blueberry [Fig 1B, 20]. After transmission to blueberry flowers, conidia adhere to the stigmatic surface, then enter the stylar canal and ovaries in a manner similar to pollen tube growth [26, 27]. Fungal hyphae colonize the fruit locules, culminating in fruit infection and the production of ‘mummified’ berries [19].

Batra and Batra (20) were the first to suggest floral mimicry in this system, finding that the brown *Mvc*-infected leaves reflect UV light and therefore may provide a visual contrast similar to flowers for pollinators. They also described a ‘fermented-tea’ odor from infected leaves, hypothesizing that the scent was attractive to vectors. Finally, they observed that some insects harbored conidia while others did not. However, the relative importance of various insect visitors (e.g., bees vs. flies) as vectors of the disease has yet to be investigated. In this paper, we address four related questions: (1) What insects visit *Mvc*-infected leaves and blueberry flowers, (2) What insects carry *Mvc* conidia and are therefore potential vectors of the disease, (3) Do *Mvc-*infected leaves mimic host floral scent, and (4) Which scent compounds are important for attraction of disease vectors?

## Methods

### Insect visitation to flowers and leaves infected with *Mvc*

All studies carried out on private land (i.e. blueberry plantings in Massachusetts, Michigan and New Jersey) were conducted with permission of the land owners. In 2008 and 2009, video observations of blueberry flowers, healthy leaves (2008 only) and leaves infected with *Monilinia vaccinii-corymbosi* (*Mvc*) were made during May and June at three mature blueberry plantings in southwest Michigan with a history of mummy berry disease (*V*. *corymbosum* cv. Jersey and Rubel). Video observations were made using bullet-type active night vision QOCDC video cameras (Q-See, Anaheim CA) mated to four channel QH25DVR digital video recorders (Q-See, Anaheim CA) and powered by a 12V deep cycle marine battery. Video equipment is fully described in Grieshop et al. [28].

In 2008, video recordings were collected from one hour before sunrise until one hour after sunset between May 15 and June 6, four days per week, which corresponded to the time of blueberry flowering and *Mvc* shoot strikes at the field sites. At each site, three cameras focused on blossom clusters, three cameras focused on *Mvc*-infected leaves, and two cameras focused on uninfected blueberry leaves. In 2009, video data were collected from one hour before sunrise until one hour after sunset from four *Mvc*-infected leaves and four flowers at each site from May 14 through June 9, seven days per week. Cameras were positioned within 15 cm of their respective target tissues and equipped with 10x macro lenses to limit the field of view and improve resolution. In both years, cameras were checked each day and moved to fresh flowers, uninfected leaves and infected leaves as required.

Video data were returned to the laboratory and evaluated by observers viewing four video frames at a time at 4x real time. More than 20,000 hours of video footage of flowers, *Mvc* shoot strikes and healthy foliage were collected, yielding 1,076 and 2,404 clips of insects interacting with their experimental subjects in 2008 and 2009, respectively. Observers recorded the identity of the insect (to order, family or species), time spent in the frame, whether the insect touched the target plant tissue, and time spent interacting with the target plant tissue. A short video clip was extracted for each behavioral event to allow later review to confirm insect identifications, contacts and duration of interactions.

### Molecular detection of *Mvc* DNA on insect visitors

To assay for the presence of *Mvc* asexual spores (conidia) on the bodies of insects, a PCR technique was designed and validated using various fungal and insect species. First, internal transcribed spacer (ITS) sequences of related *Monilinia* species were obtained from GenBank and aligned using the software package Geneious version 4.7.6 (Biomatters Ltd., Auckland, New Zealand). DNA from a variety of fungal species (*Alternaria* spp., *Botrytis cinerea*, *Colletotrichum acutatum*, *Monilinia fructicola*, *Monilinia laxa*, *Monilinia vaccinii-corymbosi*) was extracted using the phenol:chloroform method as described by Hamelin et al. [29]. Specific primers were designed for *Mvc* (MVCF and MVCR) and were tested on extracted DNA to ensure specificity (S1 and S2 Tables).

To enable detection of low amounts of *Mvc*, a nested PCR protocol was developed utilizing two sets of primers. This method was based on a two-step approach: in the first round of amplification, universal primers (ITS1F and ITS4) were used to enrich the amount of fungal DNA present in the sample. Products from the first round were then used as the template for the second round, with the species-specific primers MVCF and MVCR included in a nested reaction. The first round of PCR reactions was carried out in 25 μl of total volume consisting of 1 μl of DNA dilution (template) and 24 μl of PCR reaction mixture as described above, with the primers ITS1 and ITS4 (S1 Fig). The amplification protocol for the first step included an initial denaturation at 94°C for 2 min, followed by 30 cycles at 94°C for 1 min, 55°C for 1 min, and 72°C for 1.5 min. The reaction was completed by a 10-min extension at 72°C. The first round of PCR (1:10 dilution of 1 μl) was used as the template for the second round. PCR reactions were carried out in a total volume of 25 μl consisting of the PCR reaction mixture as described above with primers MVCF and MVCR. The amplification protocol for the second step included an initial denaturation at 94°C for 2 min, followed by 30 cycles at 94°C for 1 min, 62°C for 1 min, and 72°C for 1.5 min. The reaction was completed by a 10-min extension at 72°C. Assays were tested for sensitivity using a range of concentrations of purified genomic DNA from a laboratory *Mvc* culture which was isolated from discharged ascospores in Grand Junction, MI in 2008 (S2 Fig).

For field samples, insects were captured within three different blueberry plantings using live netting and immediately placed in kill jars from May 18 to June 5, 2009. Sampling occurred randomly in the blueberry planting by walking up and down rows and netting insects when they were found. Insects were brought into the laboratory for identification and stored at -20°C. Following visual identification of the insects (see S5 Table), the nested PCR protocol described above was used to determine the presence of *Mvc* DNA on bodies of each individual insect specimen. DNA extractions were carried out using a similar approach as described above, using between 100 to 500 mg of flash-frozen insect tissue. Following amplification, PCR fragments were separated on a 1.5% TAE-agarose gel (S3 Fig). Positive *Mvc* PCR products were sequenced using nested secondary amplification primers and BLAST analysis was used to confirm positive detections. Five of these sequences were uploaded to GenBank for future reference.

### Quantification of volatiles from flowers, healthy leaves, and leaves infected with *Mvc*

Volatile organic compounds (VOCs) were collected from highbush blueberry (*V*. *corymbosum* cv. Duke) flowers, healthy leaves, and leaves infected with *Mvc* between May 27–31, 2013. Sampling occurred on randomly chosen plants on a farm in Whately, Massachusetts between 0830–1300 h. On each day, 2 to 3 samples were collected directly from each plant tissue on mature blueberry bushes using battery-powered vacuum pumps. Air was pulled from oven bags (25 × 15 cm) that loosely enclosed each tissue type at 1 L min^−1^ for 3 hr through a filter trap containing 30 mg of a Super-Q adsorbent (Analytical Research Systems, Inc.). Each sample was taken from a cluster of 4 to 8 flowers, 6 to 13 healthy leaves, 1 to 2 infected leaves or an empty oven bag (air control). Samples were immediately transported to the laboratory and eluted with dichloromethane (150 *μ*l) containing 400 ng of *n*-octane (Sigma-Aldrich, St. Louis, MO) as an internal standard. Flowers, healthy leaves, and infected leaves (*n* = 10 for each tissue or air control) were collected, dried, and weighed to control for mass differences among samples and tissues. Compounds were separated and quantified on a Hewlett-Packard 6890 Series gas chromatograph (GC) equipped with a flame ionization detector (FID) and an Agilent HP-1 column (10 m by 0.53 mm by 2.65 *μ*m), using He as the carrier gas (constant flow = 5 ml/min, velocity = 39 cm/s). The temperature program was 40°C for 1 min, then raised at a rate of 14°C/min to 180°C, where it was held for 2 min, then raised again at a rate of 40°C/min to 200°C, where it was maintained for 2 min [30]. Individual compounds were quantified as ng/g dry material/h based on comparisons of peak areas from GC-FID with the internal standard, controlling for tissue mass of each sample. Air control samples did not contain quantifiable amounts of any compounds assessed; therefore no background corrections were necessary for the samples.

Individual compounds were identified based on retention index, referring to previous literature for blueberry leaf and flower VOCs [30, 31]. In addition, unknown compounds were characterized using a Varian 3400 gas chromatograph (Varian Inc, Palo Alto, CA) coupled with a Finnigan MAT 8230 mass spectrometer (MS; Finnigan MAT, Bremen, Germany), equipped with a Supelco MDN-5S column (30 m by 0.32 mm by 0.25 *μ*m), with He as the carrier gas. The temperature program was 35°C for 1 min, 4°C/min to 170°C, and 15°C/min to 280°C. MS data were acquired and processed in a Finnigan MAT SS300 data system. Compounds were identified by comparison of spectral data with those from the NIST library and by the GC retention index [32, 33] and confirmed by comparing their retention times with those of commercially available compounds.

We tested for multivariate differences in VOC profiles among flowers, healthy leaves, and leaves infected with *Mvc* via permutational multivariate analysis of variance (pMANOVA) using the Bray-Curtis distance matrix (100 permutations) in the package vegan in R (R Core Team 2015). We used individual VOC concentrations as abundance data, testing for acceptable heterogeneity among groups via the betadisper function (*F*_2,28_ = 4.2, *P* = 0.76). One sample of an infected leaf was excluded from analyses since an herbivorous caterpillar that was actively damaging the plant was inadvertently included in the VOC trapping bag during sampling. Thus, 10 samples from flowers, 10 samples from healthy leaves, and 9 samples from infected leaves were analyzed. Because our multivariate analysis was highly significant (*F*_2,28_ = 4.2, *P* = 0.009), we proceeded with univariate comparisons of individual compounds using restricted maximum likelihood (REML) to estimate variance parameters in linear mixed models (LMMs) with sampling date as a random effect (JMP Pro v. 11.0.0). The data met assumptions of normality and homoscedasticity. For each significant LMM, we tested for differences among tissues via Tukey’s post-hoc contrasts.

### Attraction of insects to floral and *Mvc* volatiles

During May-June 2014, we manipulated individual and synthetic blends of volatile compounds to assess their attractiveness to potential vectors of *Mvc*. We placed Delta traps (ISCA Technologies, Riverside, CA) containing volatile lures in five blueberry plantings in each of three states: Massachusetts, Michigan and New Jersey (GPS coordinates and planting descriptions in S3 Table). In each planting, traps containing one of eight treatments (seven volatile lures or a blank) were deployed for three weeks, which coincided with blueberry flowering and *Mvc* symptom development at each site. Each week, old traps were replaced with new traps with new lures to ensure consistent treatment delivery. Thus, we collected data from 360 traps (15 blueberry plantings × 8 treatments × 3 trap changes). Traps were hung in the bottom half of blueberry bushes to mimic typical spatial positioning of mummy berry shoot strikes and were randomized 10 m apart in the second row of bushes facing wooded/natural areas in each planting.

Our seven volatile lure treatments consisted of four individual volatile compounds (cinnamyl alcohol, cinnamic aldehyde, α-pinene, and 3-octen-2-one), a blend of cinnamyl alcohol and cinnamic aldehyde, a blend of α-pinene and 3-octen-2-one, and a blend of all four compounds, which were compared to a control blank. Three compounds exhibited significantly greater emission from flowers and pseudoflowers compared to healthy leaves: cinnamic aldehyde, cis-3-hexenyl methylbutyrate and linalool oxide II (Table 1). At the time of our manipulative experiment, only cinnamic aldehyde was available commercially for purchase. In addition to this compound, we selected three other commercially available compounds that were present in leaves infected by *Mvc* and in flowers, but either absent or in low abundance (α-pinene) in healthy leaves (Table 1). Cinnamyl alcohol and cinnamic aldehyde (MP Biomedicals, Santa Ana, CA) were two of the dominant scents from blueberry flowers, while α-pinene (Sigma-Aldrich, St. Louis, MO) was a dominant scent from leaves infected by *Mvc*. Similarly, 3-octen-2-one (Sigma-Aldrich) was found in leaves infected by *Mvc*, but occurred in low abundance in flowers. Thus, two of the four compounds were more abundant in flowers, while the other two were more abundant in *Mvc*-infected leaves.

**Table 1:** Concentrations of volatile compounds quantified from *Vaccinium corymbosum* flowers, healthy leaves, and leaves infected with *Monilinia vaccinii-corymbosi* (*Mvc*).

*Compound*^*1*^	*Kovats Index*^*3*^	*Mean concentration (SD)* ^†^			*F statistic*	*P value*
		*Flowers*	*Healthy leaves*	*Infected leaves*		
**Hexanal**^2^	**<800**	**1.32 (0.83)**^**a**^	**0.36 (0.39)**^**a**^	**4.14 (3.75)**^**b**^	**7.9**	**0.002**
Cis-3-hexenol^2^	811	0.81 (0.74)	0.47 (0.37)	8.13 (15.44)	2.4	0.11
α-Pinene*^,^^2^	**917**	**2.54 (1.27)**^**a**^	**0.10 (0.11)**^**a**^	**9.75 (8.58)**^**b**^	**10.1**	**<0.001**
**β-Pinene**^2^	**942**	**1.08 (1.56)**^**a**^	**0.36 (0.65)**^**a**^	**4.77 (4.78)**^**b**^	**6.5**	**0.005**
Myrcene^2^	957	0.41 (0.93)	0.17 (0.23)	0.78 (1.38)	1.0	0.38
Cis-3-hexenyl acetate^2^	965	6.44 (8.53)	1.58 (1.83)	5.45 (4.47)	2.0	0.15
Hexyl acetate^2^	970	0.62 (1.40)	0.02 (0.05)	0.39 (0.89)	1.0	0.37
**Octenone**	**989**	**1.32 (1.56)**^**ab**^	**0.05 (0.08)**^**a**^	**5.70 (5.74)**^**b**^	**4.4**	**0.010**
Limonene^2^	1003	0.00 (0.00)	0.00 (0.00)	0.96 (1.98)	2.4	0.11
Eucalyptol^2^	1011	1.10 (1.86)	0.22 (0.28)	0.57 (0.85)	1.3	0.28
**Linalool oxide I**^2^	**1046**	**0.62 (0.78)**^**ab**^	**0.05 (0.09)**^**a**^	**1.65 (2.18)**^**b**^	**3.7**	**0.039**
**Linalool oxide II**	**1056**	**1.18 (0.89)**^**b**^	**0.06 (0.08)**^**a**^	**1.71 (1.43)**^**b**^	**7.6**	**0.003**
**Linalool**^2^	**1068**	**4.60 (3.25)**^**a**^	**1.00 (0.68)**^**a**^	**10.78 (9.01)**^**b**^	**8.0**	**0.002**
*E*-4,8-Dimethyl-1,3,7-nonatriene	1098	1.98 (2.79)	0.94 (1.55)	0.97 (1.35)	0.8	0.44
3-Octen-2-one*^,^^2^	**1106**	**0.39 (1.22)**^**a**^	**0.00 (0.00)**^**a**^	**2.92 (3.31)**^**b**^	**6.0**	**0.007**
Cis-3-hexenyl propionate^2^	1121	1.17 (0.86)	0.18 (0.12)	2.24 (4.73)	1.4	0.26
Cis-3-hexenyl butyrate^2^	1149	0.43 (0.78)	0.16 (0.26)	0.00 (0.00)	1.9	0.16
**Methyl salicylate**^2^	**1170**	**3.80 (2.31)**^**a**^	**0.86 (0.78)**^**a**^	**18.27 (21.63)**^**b**^	**5.5**	**0.009**
**Benzenepropanol**	**1186**	**1.42 (1.39)**^**b**^	**0.00 (0.00)**^**a**^	**0.51 (0.78)**^**ab**^	**6.0**	**0.007**
**Cis-3-hexenyl methylbutanoate**	**1225**	**4.26 (3.76)**^**b**^	**0.10 (0.11)**^**a**^	**3.13 (2.35)**^**b**^	**7.0**	**0.004**
Cinnamic aldehyde*	**1243**	**3.55 (2.80)**^**b**^	**0.03 (0.04)**^**a**^	**4.11 (3.58)**^**b**^	**8.5**	**0.002**
Cinnamyl alcohol*^,^^2^	**1260**	**22.80 (26.67)**^**b**^	**0.00 (0.00)**^**a**^	**4.15 (5.81)**^**a**^	**5.7**	**0.009**
**Geranyl acetone**	**1310**	**0.94 (0.56)**^**a**^	**0.19 (0.17)**^**a**^	**3.09 (2.03)**^**b**^	**15.2**	**<0.001**
**β-Bourbonene**	**1323**	**1.11 (0.80)**^**a**^	**0.11 (0.18)**^**a**^	**3.85 (3.50)**^**b**^	**8.8**	**0.001**
**Cis-3-hexenyl hexanoate**^2^	**1338**	**0.59 (0.70)**^**b**^	**0.00 (0.00)**^**a**^	**0.00 (0.00)**^**a**^	**6.8**	**0.004**
β-Caryophyllene^2^	1412	0.79 (1.21)	0.41 (0.76)	1.56 (1.73)	2.0	0.16
(*E*)-β-farnesene^2^	1428	0.37 (0.81)	0.02 (0.06)	0.25 (0.76)	0.8	0.46
**(*E*,*E*)-α-farnesene**^2^	**1478**	**0.92 (1.52)**^**ab**^	**0.09 (0.17)**^**a**^	**2.43 (2.37)**^**b**^	**5.2**	**0.012**
**Total**		**66.56 (56.94)**^**ab**^	**7.52 (5.79)**^**a**^	**102.26 (83.53)**^**b**^	**6.7**	**0.005**

^1^ Compound identification was performed on a gas chromatograph coupled with a mass spectrometer (GC-MS) by comparison of spectral data with those from the NIST library and by the GC retention index.

^2^ Identification of these compounds was confirmed by matching their retention times with commercially available standards.

^3^ Kovats index as determined by [53].

^†^ Concentrations expressed as ng *n*-octane equivalents per g tissue dry mass per 3 hr sample. *F* statistic and *P* values correspond to LMM results for each compound (**bold** indicates significant LMM). Superscript letters indicate tissues that differ significantly via Tukey’s post-hoc contrasts.

* Shaded compounds were manipulated in Delta traps for the follow-up experiment (see Methods: *Attraction of insects to manipulated volatiles*, Fig 5 and S4 Fig).

Individual compounds were manipulated by adding 3 g of pure compound to 3 mL white dropping bottles (Wheaton, Millville, NJ), while blends were made by adding 3 g containing the specific ratio of compounds observed from our previously collected data on mummy berry VOCs (Table 1). The four-compound blend was created by mixing cinnamyl alcohol:cinnamic aldehyde:α-pinene:3-octen-2-one in a ratio of 20:20:47:14 by weight, which corresponds to the ratio of these compounds emitted from *Mvc* shoot strikes (Table 1). Similarly, the cinnamyl alcohol:cinnamic aldehyde blend was 50:50 by weight, and the α-pinene:3-octen-2-one blend was 77:23 by weight. Lures were separated by treatment and kept frozen until deployment in the field. Upon deployment, each lure was punctured three times with a pin at the top of the bottle, then placed upright in its respective Delta trap. Diffusion rates for each treatment were estimated by measuring the mass loss of three replicate lures per treatment for a period of one week (S4 Table).

Traps were collected weekly and all arthropods were classified to order (Arachnida, Coleoptera, Diptera, Hemiptera, Hymenoptera, Lepidoptera or other). We found no differences in arthropod abundance among the three sampling weeks (*P* > 0.05 in each case). Thus, weekly data were summed to obtain total abundances of each order for each treatment at each location. We tested for differences in arthropod abundance among treatments via linear mixed models (LMMs) with state and blueberry planting as random effects. We log-transformed all abundance data to improve normality of the residuals.

## Results

### Insect visitation to flowers and leaves infected with *Mvc*

**Fig 2:**
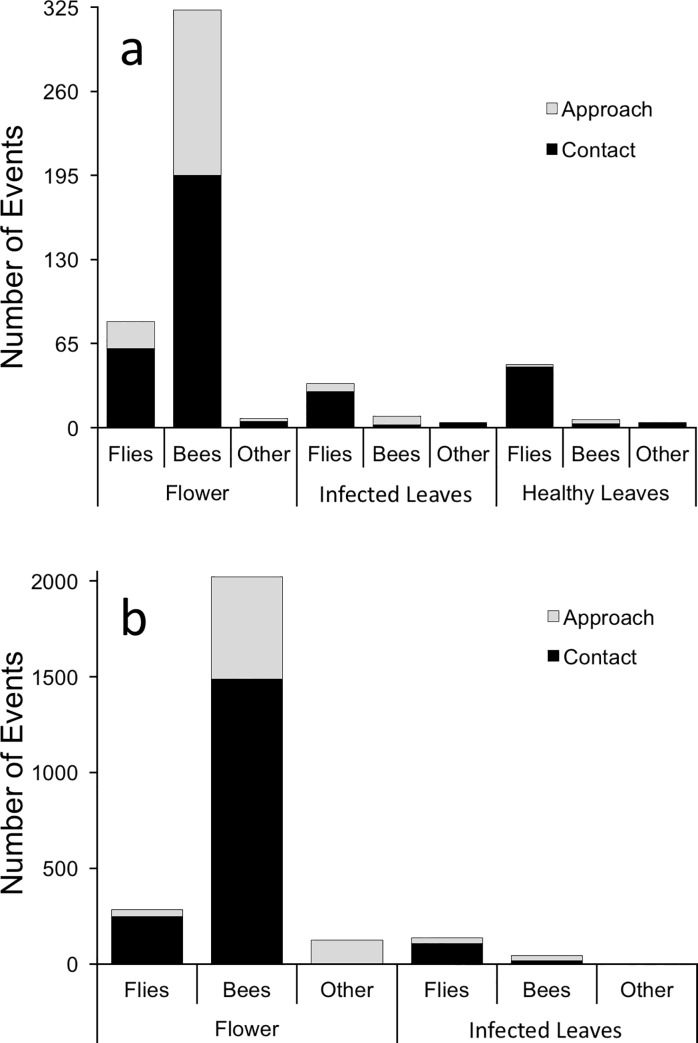
**Number of contacts (approaches that led to contacting each respective tissue) and approaches by Diptera (flies), Hymenoptera (bees) and other insects to blueberry flowers, healthy leaves, and leaves infected with *Monilinia vaccinii-corymbosi* (*Mvc*) in three Michigan blueberry plantings in 2008 (a) and 2009 (b) as observed in video-recordings.** The difference between flower vs. *Mvc*-infected leaf contacts for Hymenoptera (Bees) vs. Diptera (Flies) was significant in both years (2008: Pearson *χ*^2^ = 60.5, *P* < 0.001; 2009: Pearson *χ*^2^ = 392.7, *P* < 0.001).

Video data indicated that bees, including honey bees, bumble bees and native solitary bees, most frequently contacted flowers, making up 75–80% of total flower visits each year (Fig 2). Flies, including syrphids, muscoids, mosquitos and gnats were the next most common floral visitors, making up 14–23% of total flower visits. Flies more commonly contacted *Mvc*-infected leaves compared to bees (Fig 2), with the remainder of contacts made by ants, beetles, moths, true bugs and other insects. The difference between flower vs. *Mvc*-infected leaf contacts for bees vs. flies was significant in both years (2008: Pearson *χ*^2^ = 60.5, *P* < 0.001; 2009: Pearson *χ*^2^ = 392.7, *P* < 0.001).

### Molecular detection of *Mvc* DNA on insect visitors

**Fig 3:**
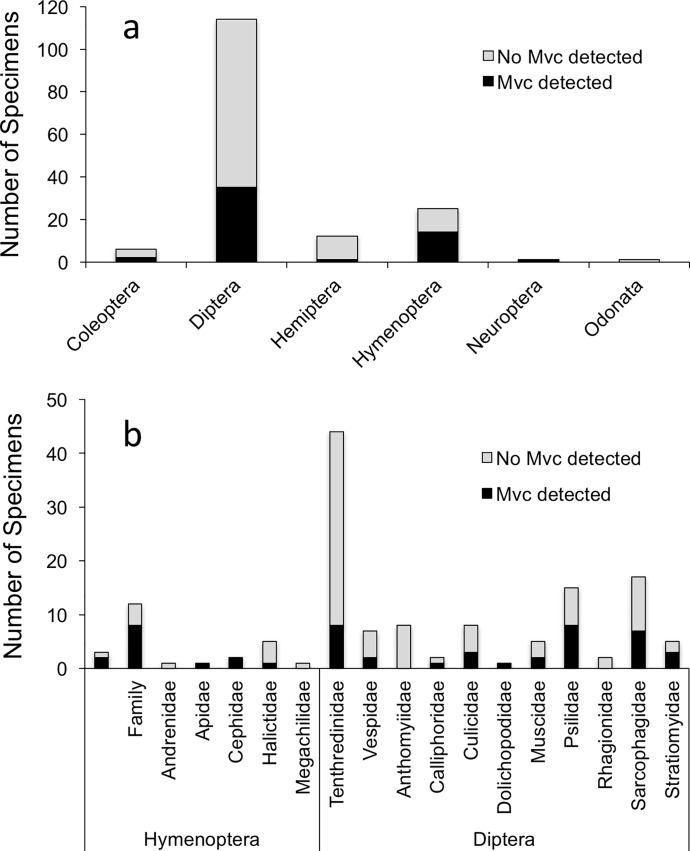
Summary of nested PCR detection of *Monilinia vaccinii-corymbosi* (*Mvc*) on insect bodies collected during 2009 (May 18^th^–June 4^th^) in three Michigan blueberry plantings. Data are summarized by insect orders sampled (a) and family for Hymenoptera and Diptera (b). The number of insects sampled for PCR detection totaled 159. A higher percentage of Hymenoptera tested positive for *Mvc* compared to Diptera (56% vs. 31%, respectively; Pearson *χ*^2^ = 5.7, *P* = 0.017).

In 2009, 159 live insects from 28 families were captured, identified and assayed using nested PCR analysis to confirm the presence or absence of *Mvc* DNA on heads and forelegs. PCR analysis found *Mvc* DNA on five of the six insect orders and eighteen of the twenty-eight insect families assayed (Fig 3A, S5 Table). DNA was found most frequently on Hymenopterans and Dipterans, with a higher percentage of bees and wasps testing positive for *Mvc* compared to flies (56% vs. 31%, respectively; Pearson *χ*^2^ = 5.7, *P* = 0.017, Fig 3B). Overall, 33% of insects captured tested positive for *Mvc* DNA (S5 Table). BLAST analysis of PCR amplification products showed that all 53 *Mvc-*positive insect samples shared a 99% maximum identity with ITS regions of *Monilinia* spp.

### Quantification of volatiles from flowers, healthy leaves, and leaves infected with *Mvc*

**Fig 4:**
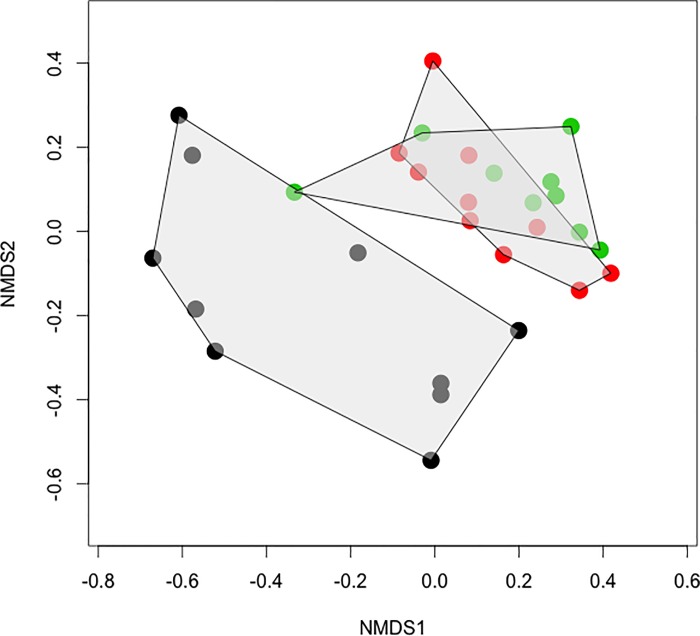
Host floral scent mimicry by *Monilinia vaccinii-corymbosi* (*Mvc*) shoot strikes. Non-metric multidimensional scaling (NMDS) plot of volatile profiles from *Vaccinium corymbosum* flowers (red points), uninfected leaves (black), and leaves infected with *Mvc* (green). Total area occupied by each sample type as represented in 2-dimensional space is shaded in gray. Stress = 0.134. VOC profiles differed significantly among the three tissues sampled (pMANOVA *F*_2,28_ = 8.1, *P* = 0.009), with healthy leaves differing from flowers (*F*_1,19_ = 9.8, *P* = 0.009) and infected leaves (*F*_1,18_ = 9.3, *P* = 0.010), while flowers vs. infected leaves were marginally, but not significantly different (*F*_1,18_ = 3.0, *P* = 0.083).

VOC profiles were significantly different among the three tissues sampled (pMANOVA *F*_2,28_ = 8.1, *P* = 0.009, Fig 4). Pair-wise comparisons among each tissue revealed that VOC profiles of healthy leaves were different from those of flowers (*F*_1,19_ = 9.8, *P* = 0.009) and infected leaves (*F*_1,18_ = 9.3, *P* = 0.010), while the profiles of flowers vs. infected leaves were marginally, but not significantly different (*F*_1,18_ = 3.0, *P* = 0.083). Concentrations of 17 of the 28 volatile compounds quantified differed significantly among tissues (Table 1), which was unlikely due to chance (binomial expansion test: *P* < 0.001). Overall, compound concentrations were highest in infected leaves, moderate in flowers and lowest in healthy leaves (Table 1; *F*_2,25_ = 6.7, *P* = 0.005).

### Attraction of insects to floral and *Mvc* volatiles

**Fig 5:**
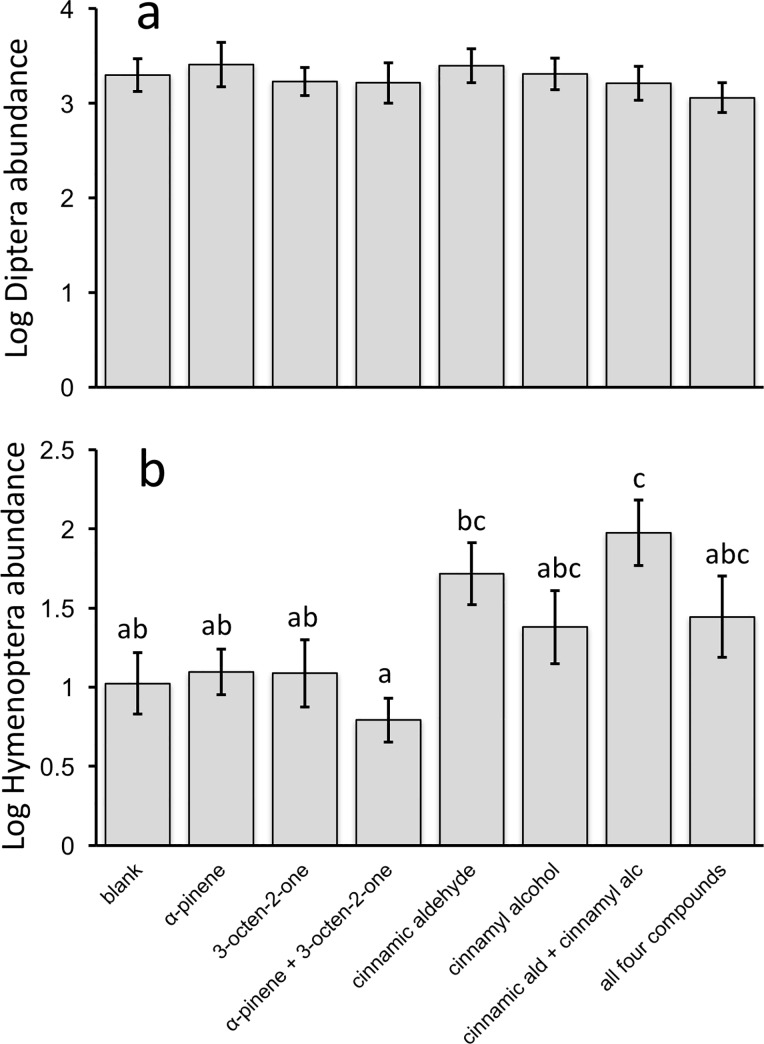
Attraction of Diptera (a) and Hymenoptera (b) to individual volatiles and synthetic blends of compounds from blueberry flowers and *Monilinia vaccinii-corymbosi* (*Mvc*) shoot strikes. Data are from five blueberry plantings in each of three states (Massachusetts, Michigan and New Jersey; *n* = 15 plantings total). Diptera did not respond to the volatile treatments (*F*_7,112_ = 0.4, *P* = 0.9), however, the abundance of Hymenoptera was affected by the treatments (*F*_7,112_ = 4.2, *P* < 0.001). Different letters correspond to treatments that are significantly different via Tukey’s post-hoc contrasts (α = 0.05). Means ± SE shown.

In total, we found 6,524 arthropods in the volatile lure Delta traps. Diptera were the most abundant insect order, comprising 64% of individuals, followed by Lepidoptera (12%), Hymenoptera (9%), Coleoptera (7%), Hemiptera (4%) and arachnids (2%). While there was substantial variation in the total abundance of arthropods among blueberry plantings (*F*_14,105_ = 10.6, *P* < 0.001), total abundance did not differ among Massachusetts, Michigan and New Jersey (*F*_2,117_ = 0.2, *P* = 0.8). We found no overall difference in total arthropod abundance among treatments (*F*_7,112_ = 0.6, *P* = 0.7), largely because Diptera did not respond to the treatments (*F*_7,112_ = 0.4, *P* = 0.9, Fig 5A). However, the abundance of Hymenoptera was affected by treatments (*F*_7,112_ = 4.2, *P* < 0.001, Fig 5B) and there were trends for differences in both Lepidoptera (*F*_7,112_ = 1.9, *P* = 0.075, S4A Fig) and Coleoptera (*F*_7,112_ = 1.9, *P* = 0.073, S4B Fig). Furthermore, the patterns among treatments were similar among Hymenoptera, Lepidoptera and Coleoptera. The combination of cinnamic aldehyde and cinnamyl alcohol was more attractive to Hymenoptera (bees and wasps) than the control blank (*P* < 0.05: Tukey’s post-hoc contrast, Fig 5B), and this general pattern was similar for both Lepidoptera and Coleoptera (S4 Fig). Cinnamic aldehyde and cinnamyl alcohol had the lowest diffusion rates of all treatments (S4 Table), indicating that increased attraction to these lures was not simply due to a greater quantity of volatile emission. All lures had greater than half of their content remaining at weekly swaps.

## Discussion

In this study we found that a diverse suite of insects interacted with blueberry flowers and leaves infected with *Monilinia vaccinii-corymbosi* (*Mvc*). A portion of the insects also harbored *Mvc* DNA on their bodies, and therefore are potential vectors of mummy berry disease. While we detected the fungus on insects across eighteen families and five orders, our data suggest that bees and flies are the main disease vectors. We also found that leaves infected with *Mvc* mimicked blueberry floral scent. The production of two dominant floral volatiles by *Mvc*-infected leaves, cinnamyl alcohol and cinnamic aldehyde, increased attraction of bees, suggesting the potential for floral volatile mimicry to enhance disease transmission between infected leaves and flowers. Yet our video data did not show increased attraction of bees or flies to leaves infected with *Mvc* compared to healthy leaves. Thus, the majority of our data, though not all, support the hypothesis that *Mvc* has evolved floral scent mimicry to facilitate asexual spore transmission via the most effective vectors in the flower-visiting community [34]. These findings complement previous studies that have investigated how unique non-host volatiles facilitate insect-mediated sexual outcrossing of fungi between pseudoflowers while minimizing vector co-visitation with host flowers [14, 16].

A major result from this study is that flies and especially bees are likely the major vectors of *Mvc*. Together, bees and flies represented 98% of contacts with blueberry flowers and 88% of contacts with shoot strikes. Bees and flies also represented 90% of positive detections of *Mvc* via PCR. Interestingly, while flies were 7.5 times more likely to visit *Mvc* shoot strikes than bees, a greater proportion of bees than flies tested positive for *Mvc* DNA. We suggest that this discrepancy between visitation patterns and *Mvc* spore loads may be due to two factors. First, the morphology of bees, specifically their abundance of branched hairs, is ideal for collecting pollen (and potentially *Mvc* spores, which are similar in morphology). In comparison, flies lack branched hairs. Thus, bees may be more likely than flies to acquire and retain spores on their bodies when visiting *Mvc-*infected leaves. Second, once *Mvc* spores have been transferred to flowers, bees may be more efficient at acquiring and dispersing spores among flowers due to their morphology and behaviors (e.g., buzz pollination), in addition to their sheer abundance as blueberry floral visitors (79% of all flower visits in this study).

Further experiments that control visitation of different insect community members at flowers and *Mvc*-infected leaves could more rigorously test the relative importance of various insects as disease transmitters. For example, in a preliminary experiment with bumble bees (*Bombus impatiens*) that were placed directly on shoot strikes and then allowed to forage on caged blueberry plants, we found that 50% of the bees (*n* = 8) transmitted spores that resulted in infection, and 54% of the flowers visited by these bees became infected (Grieshop, unpublished data). A broader array of such experiments would not only inform fundamental questions concerning the transmission ecology and vector biology of *Mvc*, but could also be useful for pollination management decisions. For example, both honey bees and bumble bees are managed during blueberry pollination. Thus, knowing the relative efficacy of these species as pollinators vs. disease vectors may be of practical importance.

Insects are essential for *Vaccinium* pollination [35–37] and the majority of *Vaccinium* pollination services are provided by wild and managed bees [38–40]. Our finding that bees were most likely to carry *Mvc* spores suggests that this important pollination service may come at a cost of disease transmission. Although flies did not visit flowers or carry spores as often as bees, due to their abundance they may still be important vectors of *Mvc*, while contributing little to pollination. Under these hypotheses, we attempted to understand whether certain *Mvc*-induced volatiles were attractive to flies vs. bees. Such knowledge could have practical importance, for example in the development of a Diptera-specific lure that could trap *Mvc* vectors while interfering minimally with blueberry pollination.

We constructed seven different lures to test this taxon-specific volatile cue hypothesis: four lures composed of individual volatiles as well as three different synthetic blends of compounds. We found that bees and wasps were attracted to volatiles produced in greater abundance by blueberry flowers than *Mvc*-infected leaves, particularly cinnamyl alcohol and cinnamic aldehyde (Fig 5B). This general pattern was also found for moths and beetles, though results were not significant (S4 Fig). Conversely, flies did not respond to any of our volatile lures (Fig 5A). In other systems, floral volatiles are important for attraction of flies (e.g. [41, 42, 43]) as well as bees (e.g. [44, 45, 46]) indicating we may not have manipulated the particular volatile components or blends attractive to flies in this system. Alternatively, although we constructed lures using the compound proportions we found in our observational data (Table 1), emission rates varied by compound (S4 Table). Thus, the relative proportions of volatiles emitted from our lures may not have matched the exact proportions emitted by *Mvc*-infected shoots, which may have influenced insect attraction. Regardless, our data does allow us to test whether presence/absence of individual and combinations of compounds impacted attraction among a diverse community of insects, including flies. Given increased interest in Integrated Pest Management strategies for pest management by berry producers, further exploration into taxa-specific lures and traps could be fruitful.

The diversity of volatiles induced by *Mvc* infection provides insight into host plant defensive responses against *Mvc* as well as floral mimicry and vector attraction. For example, the two dominant compounds that were emitted from *Mvc*-infected leaves were α-pinene and methyl salicylate (Table 1). α-pinene is known to be used by plants as an antimicrobial defense against fungal plant pathogens in other systems (e.g. [47, 48]). In addition, the conversion of salicylic acid to derivatives such as salicylic acid glucose ester and methyl salicylate, are general plant responses to pathogen infection [49, 50]. Interestingly, when citrus plants are infected with ‘*Candidatus* Liberibacter asiaticus,’ the bacteria causing citrus greening disease, methyl salicylate is also a dominant volatile that is induced [4]. Psyllids, the main vectors of the disease, are attracted to the induced methyl salicylate from infected plants [4], suggesting volatiles associated with plant defense can also be used as cues by disease vectors.

While our data indicate floral volatile mimicry by *Mvc* (Fig 4) and attraction of potential disease vectors to specific induced volatiles (cinnamyl alcohol and cinnamic aldehyde; Fig 5), our video data suggest similar attraction of insects to *Mvc*-infected leaves compared to healthy leaves (Fig 2A). This apparent discrepancy may be an artifact of several factors, for example the different varieties used in the video experiment (*V*. *corymbosum* cv. Jersey and Rubel) vs. volatile collection experiment (*V*. *corymbosum* cv. Duke), or perhaps that attraction of insects to the brown infected leaves would be even lower than attraction to healthy leaves if odors weren’t present. Our data cannot assess these hypotheses, though each possibility raises interesting questions. For example, *Vaccinium* varieties are known to vary in herbivore-induced volatile production [51]. Perhaps genetic variation in volatile production by *Mvc*-infected leaves is partially responsible for differences in resistance to leaf and fruit infection observed among blueberry varieties, including Jersey, Rubel and Duke [52]. Further understanding of phenotypic expression of *Mvc*, in addition to the genetic underpinnings of disease induced traits, could inform both general knowledge regarding disease transmission and breeding efforts for resistance.

In summary, we have shown that leaves infected with *Mvc* mimic host floral scent, that *Mvc* DNA is present on a diverse suite of potential insect vectors, and that flies and especially bees are likely the main disease vectors. These data complement previous evidence that visual traits of *Mvc*-infected leaves may simulate blueberry flowers [20], suggesting the potential for a multimodal olfactory and visual mimicry system. Overall, our results provide insight into the evolutionary and chemical ecology of an economically important pollinator-vectored plant pathogen.

## Supporting Information

S1 Fig Diagram showing the positions of the nested, species-specific primers for *Monilinia vaccinii-corymbosi* (MVCF and MVCR) and the universal primers, ITS1F and ITS4, within the internal transcribed spacer (ITS) regions. For the nested polymerase chain reaction, products from the first round of amplification with the universal ITS1F and ITS4 primers are used as template in the second round with all of the species-specific primers in a multiplex reaction. Primer pairs MVCF-MVCR yield amplification products of 218 bp.
(TIFF)

S2 Fig Sensitivity of nested PCR for the detection of *Monilinia vaccinii-corymbosi* using purified genomic DNA. Lane 1, 1-kb+ DNA ladder; lanes 2–10, serial dilution of genomic DNA; lane 2, 100 ng; lane 3, 10 ng; lane 4, 1 ng; lane 5, 100 pg; lane 6, 10 pg; lane 7, 1 pg; lane 8, 100 fg; lane 9, 10 fg; lane 10, 1 fg; lane 11, 100 ag; lane 12, 10 ag; lane 13, 1 ag; lane 14 water control from primary PCR reaction.
(TIFF)

S3 Fig Nested PCR detection of *Monilinia vaccinii-corymbosi* using DNA extracted from insect body surfaces collected in three different blueberry plantings in Southwest Michigan. Lane 1, 1-kb+ DNA ladder; lane 2, water control; lane 3, water control from primary PCR; lanes 4–16 DNA extracted from different insect samples. Note: (+) is positive and (-) is negative for *M*. *vaccinii-corymbosi*.
(TIFF)

S4 Fig **Attraction of Lepidoptera (a) and Coleoptera (b) to individual volatiles and synthetic blends of compounds from blueberry flowers and *Monilinia vaccinii-corymbosi* (*Mvc*) shoot strikes.** Results are marginally significant for Lepidoptera (*F*_7,112_ = 1.9, *P* = 0.075) and Coleoptera (*F*_7,112_ = 1.9, *P* = 0.073). Means ± SE shown.
(TIFF)

S1 Table Sequence, guanine-cytosine (GC) percentage, and calculated melting temperature (Tm) of the primers for the ITS region (ITS1F-ITS4) and *Monilinia vaccinii-corymbosi* (MVCF-MVCR) used in polymerase chain reaction amplifications. (DOCX)

S2 Table Traditional, non-nested PCR results using genomic DNA from various fungal and oomycete genera and species occurring on blueberries and other fruit crops grown in Michigan to evaluate the specificity of primers designed for *Monilinia vaccinii-corymbosi*. Amplification by primer set; positive (+) or negative (-).
(DOCX)

S3 Table Location and description of 15 blueberry plantings where Delta traps were placed in 2014. Lures containing individual volatiles and synthetic blends of compounds from blueberry flowers and *Monilinia vaccinii-corymbosi* (*Mvc*) shoot strikes were placed in Delta traps to evaluate attractiveness of volatiles to insects.
(DOCX)

S4 Table Normalized release rate of individual volatiles and synthetic blends of compounds from blueberry flowers and *Monilinia vaccinii-corymbosi* (*Mvc*) shoot strikes that were assessed via volatile lures in Delta traps in blueberry plantings in Massachusetts, Michigan and New Jersey. (DOCX)

S5 Table Summary of nested PCR detection of *Monilinia vaccinii-corymbosi* (*Mvc*) on all insect bodies collected in 2009 (May 18^th^–June 4^th^) from three blueberry plantings in southwest Michigan. Data summarized by insect order and family.
(DOCX)
