# Supplementary figures and images for: Floral Scent Mimicry and Vector-Pathogen Associations in a Pseudoflower-Inducing Plant Pathogen System
Source: PLoS One. 2016 Nov 16;11(11):e0165761. doi: 10.1371/journal.pone.0165761 (PMC5113062; doi:10.1371/journal.pone.0165761)

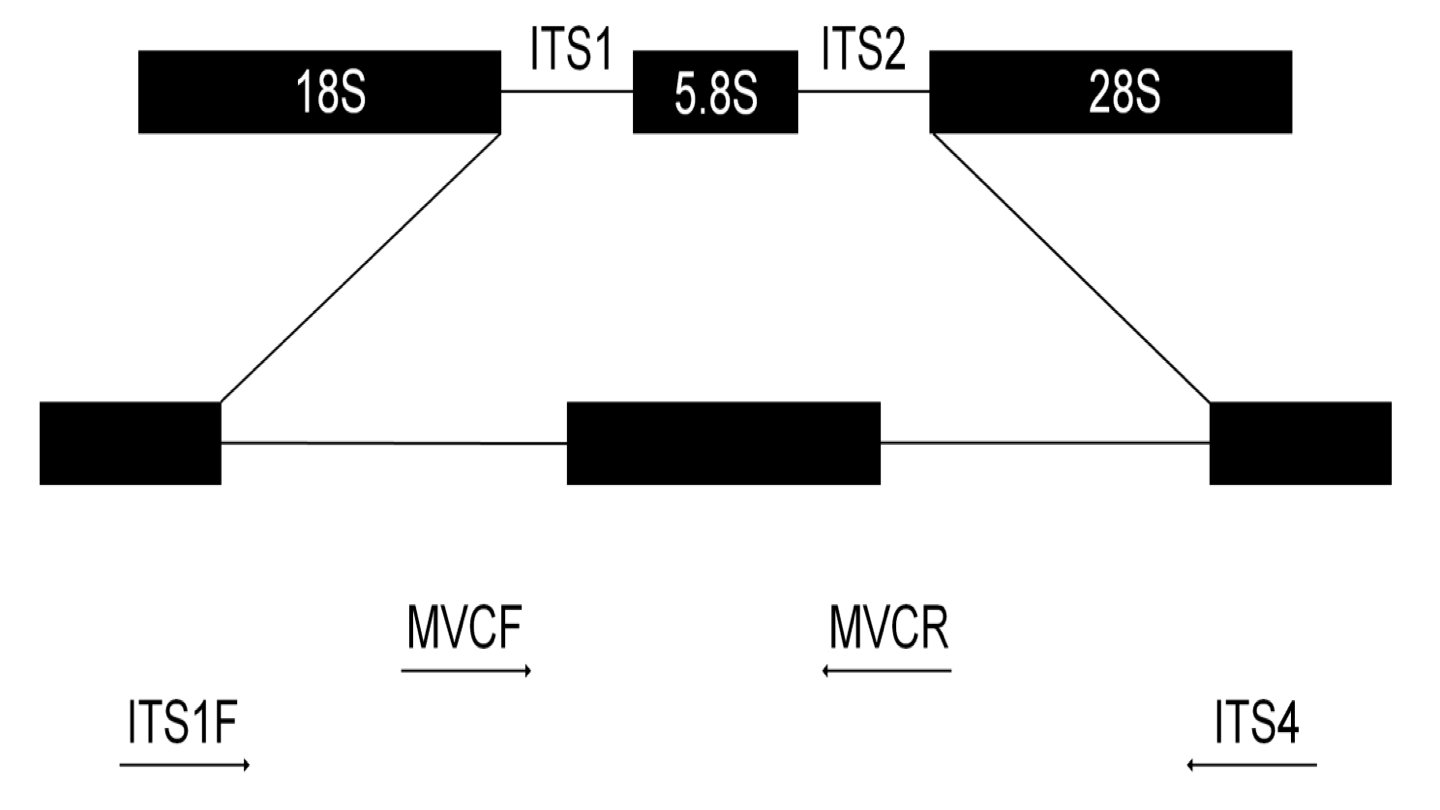

Supplement: S1 Fig — For the nested polymerase chain reaction, products from the first round of amplification with the universal ITS1F and ITS4 primers are used as template in the second round with all of the species-specific primers in a multiplex reaction. Primer pairs MVCF-MVCR yield amplification products of 218 bp. (TIFF) [file pone.0165761.s001.tiff]

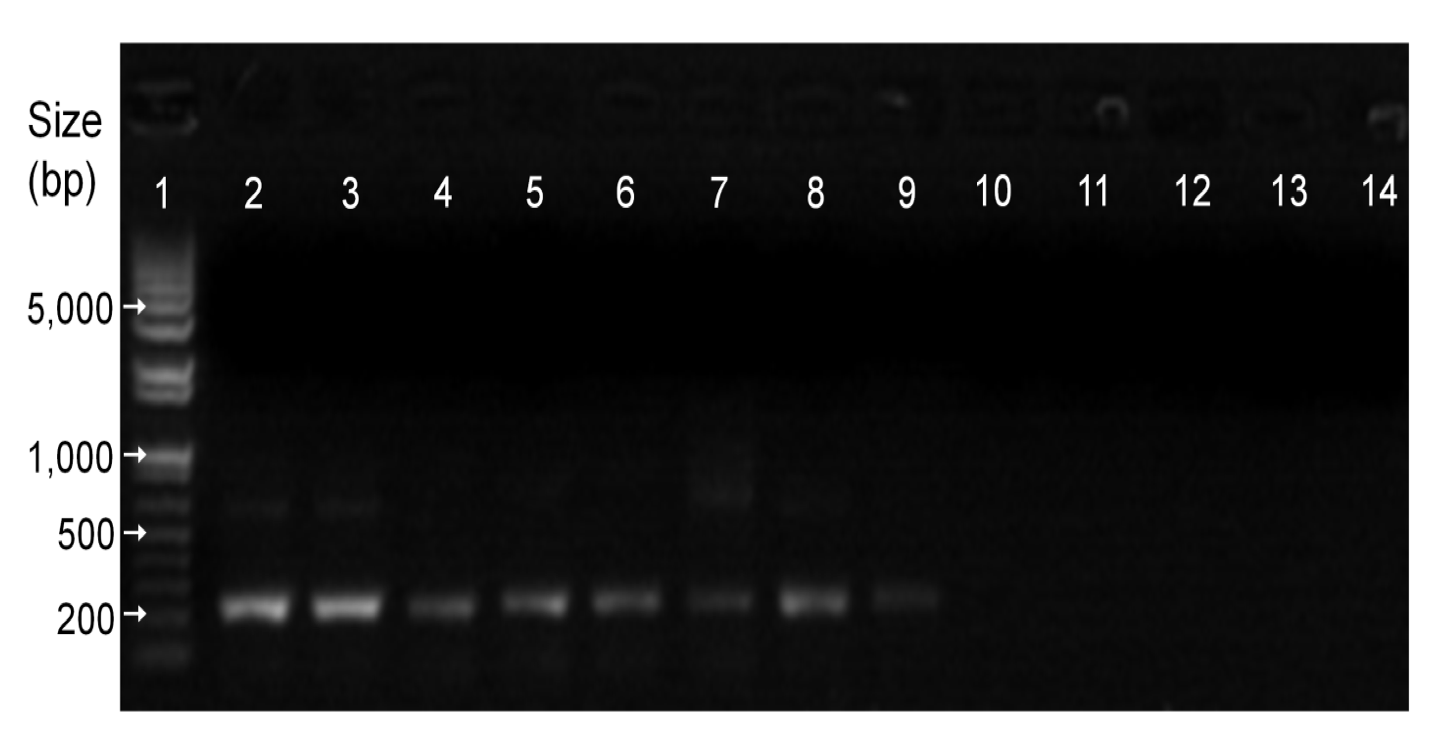

Supplement: S2 Fig — Lane 1, 1-kb+ DNA ladder; lanes 2–10, serial dilution of genomic DNA; lane 2, 100 ng; lane 3, 10 ng; lane 4, 1 ng; lane 5, 100 pg; lane 6, 10 pg; lane 7, 1 pg; lane 8, 100 fg; lane 9, 10 fg; lane 10, 1 fg; lane 11, 100 ag; lane 12, 10 ag; lane 13, 1 ag; lane 14 water control from primary PCR reaction. (TIFF) [file pone.0165761.s002.tiff]

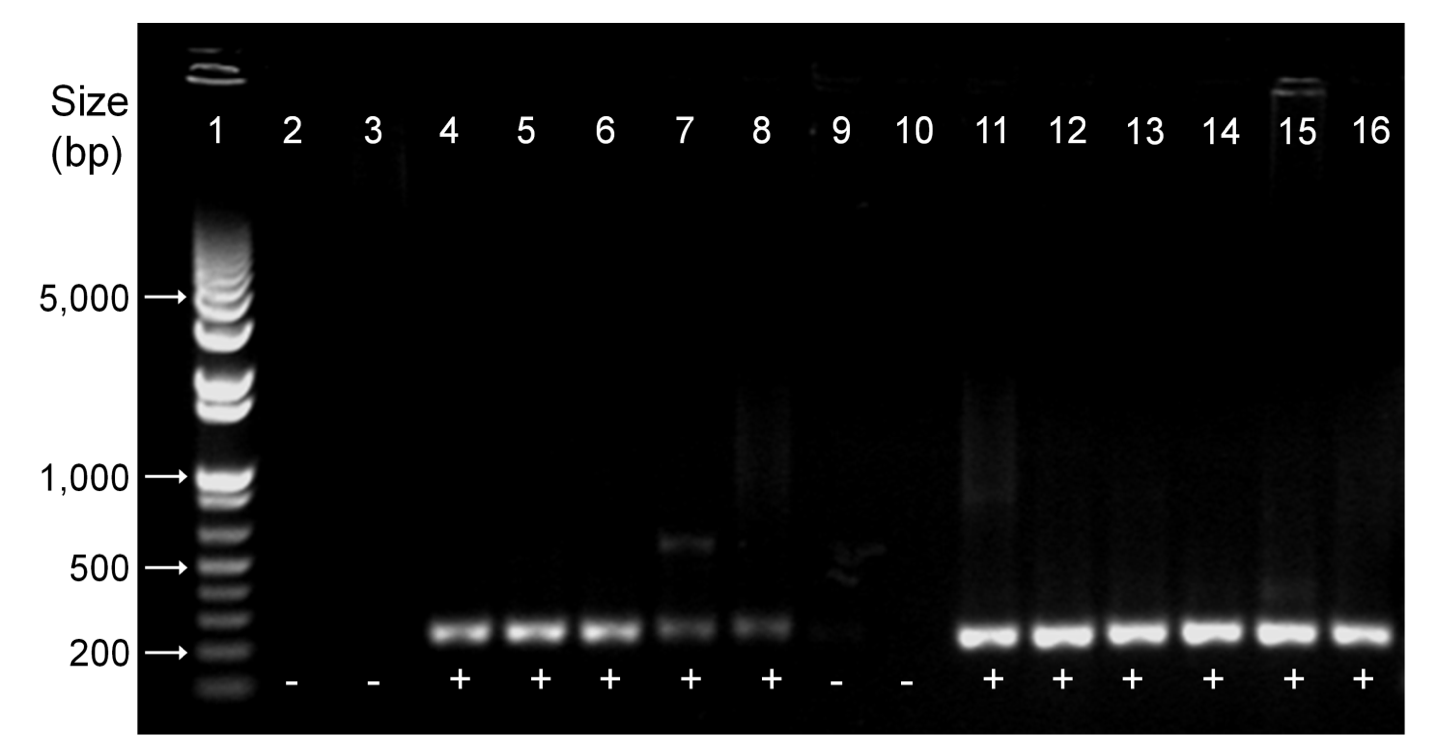

Supplement: S3 Fig — Lane 1, 1-kb+ DNA ladder; lane 2, water control; lane 3, water control from primary PCR; lanes 4–16 DNA extracted from different insect samples. Note: (+) is positive and (-) is negative for M. vaccinii-corymbosi. (TIFF) [file pone.0165761.s003.tiff]

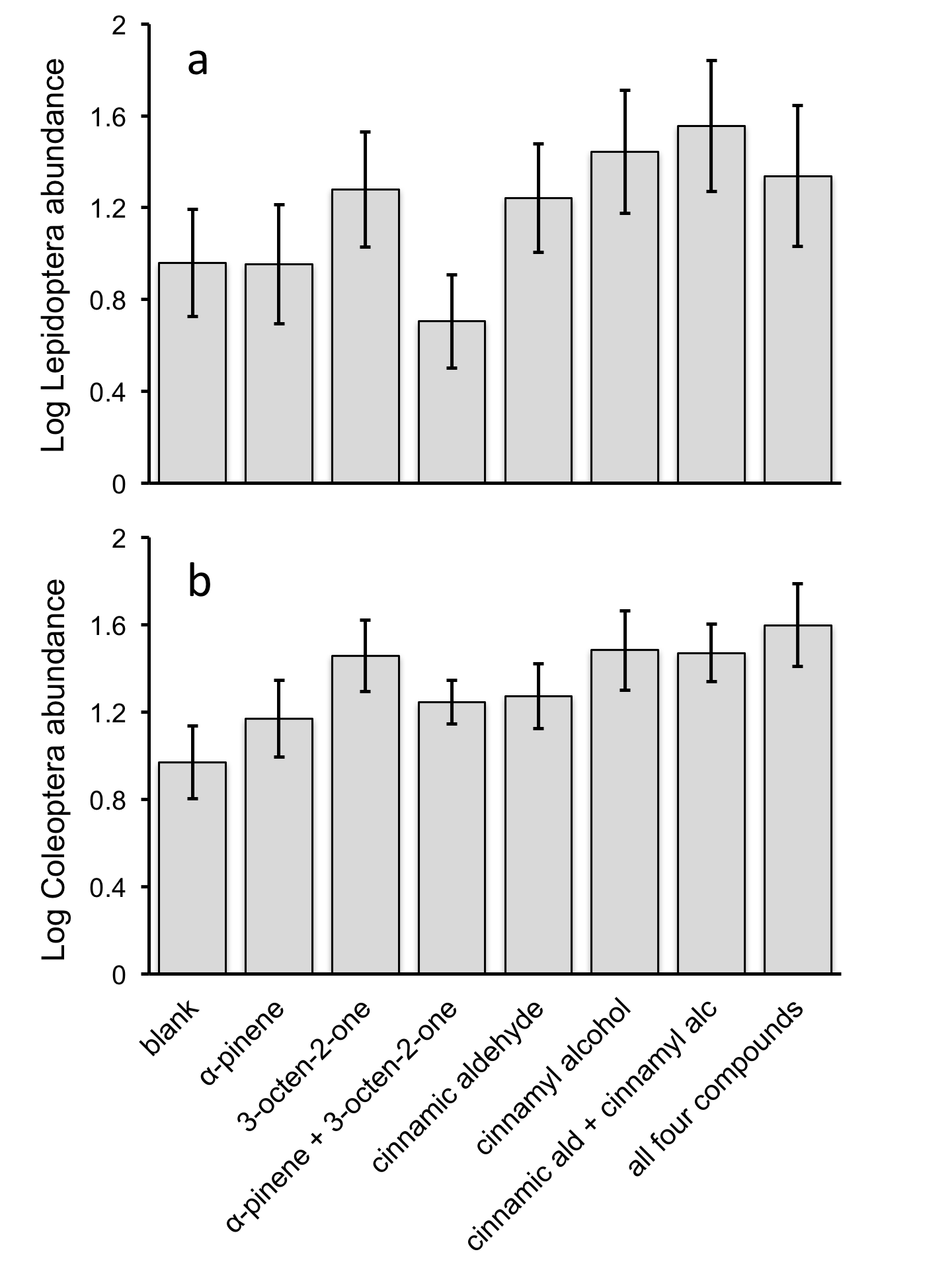

Supplement: S4 Fig — Attraction of Lepidoptera (a) and Coleoptera (b) to individual volatiles and synthetic blends of compounds from blueberry flowers and Monilinia vaccinii-corymbosi (Mvc) shoot strikes. Results are marginally significant for Lepidoptera (F7,112 = 1.9, P = 0.075) and Coleoptera (F7,112 = 1.9, P = 0.073). Means ± SE shown. (TIFF) [file pone.0165761.s004.tiff]
