# Supplementary material for: Floral Scent Mimicry and Vector-Pathogen Associations in a Pseudoflower-Inducing Plant Pathogen System
Source: PLoS One. 2016 Nov 16;11(11):e0165761. doi: 10.1371/journal.pone.0165761 (PMC5113062; doi:10.1371/journal.pone.0165761)
Supplement: S1 Table — (DOCX) [file pone.0165761.s005.docx]

**S1 Table.** Sequence, guanine-cytosine (GC) percentage, and calculated melting temperature (Tm) of the primers for the ITS region (ITS1F-ITS4) and *Monilinia vaccinii-corymbosi* (MVCF-MVCR) used in polymerase chain reaction amplifications.

| Primer | Sequence (5’-3’) | Reference | GC (%) | T_m_ | Product size (bp) |
| --- | --- | --- | --- | --- | --- |
| ITS1F | CTT GGT CAT TTA GAG GAA GTA A | Gardes and Bruns (54) | 36.4 | 49.7 | ~520 |
| ITS4 | TCC TCC GCT TAT TGA TAT GC | White, Bruns (55) | 45.0 | 52.1 |  |
| MVCF | GCC CGC CAA TGA CCT AAC | This manuscript | 61.1 | 56.6 | 218 |
| MVCR | TTG AAA TGA CGC TCG AAC AG | This manuscript | 45.0 | 53.3 |  |
