# Supplementary material for: Floral Scent Mimicry and Vector-Pathogen Associations in a Pseudoflower-Inducing Plant Pathogen System
Source: PLoS One. 2016 Nov 16;11(11):e0165761. doi: 10.1371/journal.pone.0165761 (PMC5113062; doi:10.1371/journal.pone.0165761)
Supplement: S2 Table — Amplification by primer set; positive (+) or negative (-). (DOCX) [file pone.0165761.s006.docx]

**S2 Table**. Traditional, non-nested PCR results using genomic DNA from various fungal and oomycete genera and species occurring on blueberries and other fruit crops grown in Michigan to evaluate the specificity of primers designed for *Monilinia vaccinii-corymbosi*. Amplification by primer set; positive (+) or negative (-).

| Species tested | Host | ITS1F-ITS4^1^ | MVCF-MVCR^2^ |
| --- | --- | --- | --- |
| *Alternaria* sp. | *V. corymbosum* | + | - |
| *Botrytis cinerea* | *V. corymbosum* | + | - |
| *Colletotrichum acutatum* | *V. corymbosum* | + | - |
| *Monilinia fructicola* | *Prunus* spp. | + | - |
| *Monilinia laxa* | *Prunus* spp. | + | - |
| *Monilinia vaccinii-corymbosi* | *V. corymbosum* | + | + |
| *Roesleria subterranea* | *Vitis* interspecific hybrid | + | - |
| *Phytophthora* sp. | *V. corymbosum* | - | - |
| *Phytopythium sterilum* | *V. corymbosum* | - | - |

^1^ 2 min at 94°C. then 40 cycles of 1 min at 94°C, 1 min at 55°C and 1.5 min at 72°C followed 10 min at 72°C.

^2^ 2 min at 94°C. then 40 cycles of 1 min at 94°C, 1 min at 60°C and 1.5 min at 72°C followed 10 min at 72°C. For nested reactions, only 25 cycles were used.
