# Supplementary material for: Floral Scent Mimicry and Vector-Pathogen Associations in a Pseudoflower-Inducing Plant Pathogen System
Source: PLoS One. 2016 Nov 16;11(11):e0165761. doi: 10.1371/journal.pone.0165761 (PMC5113062; doi:10.1371/journal.pone.0165761)
Supplement: S3 Table — Lures containing individual volatiles and synthetic blends of compounds from blueberry flowers and Monilinia vaccinii-corymbosi (Mvc) shoot strikes were placed in Delta traps to evaluate attractiveness of volatiles to insects. (DOCX) [file pone.0165761.s007.docx]

**S3 Table.** Location and description of 15 blueberry plantings where Delta traps were placed in 2014. Lures containing individual volatiles and synthetic blends of compounds from blueberry flowers and *Monilinia vaccinii-corymbosi* (*Mvc*) shoot strikes were placed in Delta traps to evaluate attractiveness of volatiles to insects.

| **State** | **Site** | **GPS coordinates** | **Planting size (acres)** | **Mummy berry present in planting?** |
| --- | --- | --- | --- | --- |
| Massachusetts | A | 42°26'38.9"N 72°38'17.9"W | 3.5 | Yes |
| Massachusetts | B | 42°21'56.6"N 72°49'43.0"W | 8.2 | Yes |
| Massachusetts | C | 42°25'49.4"N 72°35'18.0"W | 2.2 | No |
| Massachusetts | D | 42°15'59.1"N 72°26'42.5"W | 2.0 | Yes |
| Massachusetts | E | 42°15'04.4"N 72°21'44.0"W | 0.6 | Yes |
| Michigan | A | 42°32'58.3"N 84°54'24.6"W | 0.5 | No |
| Michigan | B | 42°32'58.3"N 84°54'24.6"W | 1.4 | Yes |
| Michigan | C | 42°28'18.6"N 84°31'15.4"W | 1.2 | Yes |
| Michigan | D | 42°28'58.2"N 84°28'40.9"W | 1.0 | Yes |
| Michigan | E | 42°32'12.7"N 84°24'22.6"W | 1.0 | Yes |
| New Jersey | A | 39°39'41.2"N  74°45'6.7"W | 9.5 | Unknown |
| New Jersey | B | 39°39'57.5"N  74°44'57.9"W | 3.5 | Unknown |
| New Jersey | C | 39°43'3.6"N  74°30'52.4"W | 6.1 | Yes |
| New Jersey | D | 39°43'1.9"N  74°30'18.9"W | 3.1 | Yes |
| New Jersey | E | 39°57'15.9"N  74°30'22.1"W | 6.8 | Unknown |
