# Supplementary material for: Floral Scent Mimicry and Vector-Pathogen Associations in a Pseudoflower-Inducing Plant Pathogen System
Source: PLoS One. 2016 Nov 16;11(11):e0165761. doi: 10.1371/journal.pone.0165761 (PMC5113062; doi:10.1371/journal.pone.0165761)
Supplement: S4 Table — (DOCX) [file pone.0165761.s008.docx]

**S4 Table.** Normalized release rate of individual volatiles and synthetic blends of compounds from blueberry flowers and *Monilinia vaccinii-corymbosi* (*Mvc*) shoot strikes that were assessed via volatile lures in Delta traps in blueberry plantings in Massachusetts, Michigan and New Jersey.*

| **Treatment** | **Change in mass (mg/d)** | **Standard deviation**  **(mg/d)** |
| --- | --- | --- |
|  |  |  |
| α-pinene | -29.71 | 4.0 |
| 3-octen-2-one | -18.97 | 2.2 |
| Cinnamic aldehyde | 0.01 | 4.3 |
| Cinnamyl alcohol | -6.66 | 1.3 |
| α-pinene and 3-octen-2-one | -42.83 | 7.5 |
| Cinnamic aldehyde and cinnamyl alcohol | -3.37 | 4.5 |
| All four compounds | -25.38 | 3.2 |
| ^*^Normalized by subtracting change in mass of blank control. | | |
|  |  |  |
