# Supplementary material for: Floral Scent Mimicry and Vector-Pathogen Associations in a Pseudoflower-Inducing Plant Pathogen System
Source: PLoS One. 2016 Nov 16;11(11):e0165761. doi: 10.1371/journal.pone.0165761 (PMC5113062; doi:10.1371/journal.pone.0165761)
Supplement: S5 Table — Data summarized by insect order and family. (DOCX) [file pone.0165761.s009.docx]

**S5 Table.** Summary of nested PCR detection of *Monilinia vaccinii-corymbosi* (*Mvc*) on all insect bodies collected in 2009 (May 18^th^ – June 4^th^) from three blueberry plantings in southwest Michigan. Data summarized by insect order and family.

| **Order** | **Family** | **Number collected** | **Positive for *Mvc*** | **Negative for *Mvc*** | **Percent positive for *Mvc*** |
| --- | --- | --- | --- | --- | --- |
| Coleoptera | Chrysomelidae | 2 | 0 | 2 | 0 |
|  | Cicindelidae | 2 | 1 | 1 | 50 |
|  | Scarabaeidae | 2 | 1 | 1 | 50 |
| Diptera | Anthomyiidae | 44 | 8 | 36 | 18 |
|  | Calliphoridae | 7 | 2 | 5 | 29 |
|  | Culicidae | 8 | 0 | 8 | 0 |
|  | Dolichopodidae | 2 | 1 | 1 | 50 |
|  | Muscidae | 8 | 3 | 5 | 38 |
|  | Psilidae | 1 | 1 | 0 | 100 |
|  | Rhagionidae | 5 | 2 | 3 | 40 |
|  | Sarcophagidae | 15 | 8 | 7 | 53 |
|  | Stratiomyidae | 2 | 0 | 2 | 0 |
|  | Syrphidae | 17 | 7 | 10 | 41 |
|  | Tabanidae | 5 | 3 | 2 | 60 |
| Hemiptera | Cercopidae | 4 | 1 | 3 | 25 |
|  | Cicadellidae | 1 | 0 | 1 | 0 |
|  | Miridae | 5 | 0 | 5 | 0 |
|  | Nabidae | 1 | 0 | 1 | 0 |
|  | Rhopalidae | 1 | 0 | 1 | 0 |
| Hymenoptera | Andrenidae | 3 | 2 | 1 | 67 |
|  | Apidae | 12 | 8 | 4 | 67 |
|  | Cephidae | 1 | 0 | 1 | 0 |
|  | Halictidae | 1 | 1 | 0 | 100 |
|  | Megachilidae | 2 | 2 | 0 | 100 |
|  | Tenthredinidae | 5 | 1 | 4 | 20 |
|  | Vespidae | 1 | 0 | 1 | 0 |
| Neuroptera | Chrysopidae | 1 | 1 | 0 | 100 |
| Odonata | Coenagrionidae | 1 | 0 | 1 | 0 |
| **Total or Mean** | | **159** | **53** | **106** | **33** |
